# Impact of Different Extraction Methods on the Physicochemical Characteristics and Bioactivity of Polysaccharides from Baobab (*Adansonia suarezensis*) Fruit Pulp

**DOI:** 10.3390/foods15020273

**Published:** 2026-01-12

**Authors:** Huimin Cui, Shang Gao, Jiahui Shi, Yinghui Pan, Pengzhi Hong, Jiannong Lu, Chunxia Zhou

**Affiliations:** 1 College of Food Science and Technology, Guangdong Ocean University, Guangdong Provincial Key Laboratory of Aquatic Product Processing and Safety, Guangdong Province Engineering Laboratory for Marine Biological Products, Guangdong Provincial Engineering Technology Research Center of Seafood, Guangdong Provincial Engineering Technology Research Center of Prefabricated Seafood Processing and Quality Control, Zhanjiang 524088, China; 13437863642@163.com (H.C.); gaos1004@163.com (S.G.); shui4357@163.com (J.S.); 15382615996@163.com (Y.P.); hongpengzhi@126.com (P.H.); 2 Southern Marine Science and Engineering Guangdong Laboratory (Zhanjiang), Zhanjiang 524088, China; 3 College of Coastal Agricultural Sciences, Guangdong Ocean University, Zhanjiang 524088, China

**Keywords:** *Adansonia suarezensis* fruit pulp, polysaccharides, structure characteristics, antioxidant activity, hypoglycemic activity

## Abstract

Polysaccharides from baobab (*Adansonia suarezensis*) fruit pulp (ASPs) hold significant potential for pharmaceutical and functional food applications due to their bioactivities. This study systematically evaluated the effects of six extraction methods—hot water (ASP-HW), acid (ASP-AC), alkaline (ASP-AL), and their ultrasound-assisted counterparts (ASP-HWU, ASP-ACU, ASP-ALU)—on the yield, chemical composition, structural properties, and biological activities of ASPs. The results demonstrated that the extraction solvent critically influenced key properties: alkaline-based methods (ASP-AL, ASP-ALU) achieved the highest yields (up to 62.47%) and yielded polysaccharides with lower molecular weights (approximately 19,600–19,813 Da) and smaller particle sizes (around 140–147 nm). All ASPs were identified as acidic pectic polysaccharides, composed of galacturonic acid, xylose, galactose, and arabinose. Notably, ASP-AC, ASP-ACU, ASP-AL, and ASP-ALU exhibited a triple-helix conformation, which was absent in hot water-extracted polysaccharides. Bioactivity assessments revealed that ASP-AL and ASP-ALU possessed superior antioxidant capacities, demonstrating the lowest IC_50_ values for DPPH radical scavenging (113.67–116.67 μg/mL) and ABTS radical scavenging (79.33–79.67 μg/mL), as well as potent α-glucosidase inhibitory activity (IC_50_: 0.146–0.206 mg/mL), outperforming other extracts and the positive control acarbose. Correlation analysis indicated that enhanced bioactivity was associated with lower molecular weight and reduced uronic acid content. These findings underscore that alkaline extraction is an efficient strategy for obtaining highly bioactive polysaccharides from *Adansonia suarezensis* fruit pulp, providing a valuable theoretical foundation for their utilization in developing nutraceuticals and functional foods.

## 1. Introduction

The genus *Adansonia*, part of the *Bombacoideae* subfamily in the *Malvaceae* family, comprises eight existing species of tall deciduous trees globally. Among these, six species are endemic to Madagascar, while the remaining two are distributed in mainland Africa and Australia, respectively [1]. All components of the baobab tree, including its fruit, leaves, seeds, roots, and bark, are valuable food sources [2]. Additionally, they are utilized in traditional medicine to treat ailments such as diarrhea, dysentery, and fever [3], highlighting their significant nutritional, medicinal, and economic importance. In 2008, the European Commission authorized dried baobab fruit pulp as a novel food, and the U.S. Food and Drug Administration followed suit in 2009, approving it as a food ingredient. The drastic decline in suitable habitats for *Adansonia* trees, caused by global climate change and habitat destruction, has attracted considerable scientific and public concern regarding their conservation. *Adansonia suarezensis*, a species indigenous to Madagascar, was listed as endangered on the IUCN Red List of Threatened Species in December 2015 [4]. Under the background, the Jubao Lin Seedling Planting Professional Cooperative introduced *Adansonia suarezensis* to Zhanjiang, Guangdong Province two decades ago. The introduced tree has now developed trunks of substantial girth, requiring approximately three adults with linked arms to encircle it. In 2023, the tree bore fruit for the first time following artificial pollination. The mature fruits are oval shaped, measuring approximately 30–35 cm in length and 15–20 cm in width. This successful introduction and fruiting demonstrate the species’ adaptability to the local climate in Zhanjiang and its responsiveness to artificial pollination.

Research indicates that natural polysaccharides exhibit a range of biological activities, including antioxidant, immunomodulatory, antiviral, antitumor, anti-aging, and hypolipidemic effects [5]. Compared to chemically synthesized drugs, polysaccharides derived from natural plant sources exhibit minimal side effects [6]. Consequently, increasing research efforts are dedicated to identifying naturally active polysaccharides capable of replacing synthetic drugs. The composition and bioactivity of baobab fruit polysaccharides may vary significantly depending on their growth environments. Previous research has shown that baobab fruit polysaccharides exhibit significant antioxidant, antihyperglycemic, antimicrobial, antihypertensive, and anti-inflammatory properties. In a study by Bougatef et al., polysaccharides extracted from baobab (*Adansonia digitata*) fruit pulp in southern Mauritania exhibited a DPPH radical scavenging rate of 97.09 ± 0.01% at 5 mg/mL, an ABTS radical scavenging rate of 96.85%, and a ferrous ion chelating capacity of 75.50%. The polysaccharides also showed significant inhibitory effects against *Salmonella enterica*, *Salmonella thyphi*, *Klebsiella pneumoniae*, *Escherichia coli*, *Micrococcus luteus*, and *Staphylococcus aureus*. Additionally, the IC_50_ value for angiotensin-I-converting enzyme inhibitory activity was determined to be 0.29 mg/mL [7]. Song et al. isolated two polysaccharides (ADPs40-F3, ADPs60-F3) from baobab (*Adansonia digitata*) fruit pulp through a process of graded ethanol precipitation. These polysaccharides demonstrated excellent antioxidant activity and in vitro hypoglycemic activity, respectively. Furthermore, the high-dose group exhibited significant anti-diabetic properties in mouse in vivo experiments [8].

The physicochemical properties and bioactivity of polysaccharides was significantly influenced by extraction method [9]. Alongside conventional methods such as hot water extraction, acid extraction, alkali extraction, ultrasound extraction, microwave extraction, and enzyme-assisted extraction, several emerging techniques—including subcritical water extraction, supercritical fluid extraction, and dynamic high-pressure microfluidization -assisted extraction—have also been used to improve the extraction yield of polysaccharides [10,11]. For instance, polysaccharides extracted from *Volvariella volvacea* using citric acid (VVP-C) exhibit stronger inhibitory activity against α-amylase and α-glucosidase compared to those extracted with water or alkali [12]. Jiang et al. compared the bioactivities of polysaccharides from *Ficus carica* L. leaves using seven different methods (hot water extraction, ultrasound-assisted extraction, alkali extraction, microwave-assisted extraction, ultrasound–microwave synergistic extraction, ultrasound-assisted enzymatic extraction, and high-speed shear homogenization extraction) and found that the polysaccharides obtained through ultrasound-assisted extraction exhibited the highest antioxidant and hypoglycemic activities [13].

To date, no studies in China have reported on the structural characteristics and bioactivities of polysaccharides from baobab fruit, particularly *Adansonia suarezensis*, which has been cultivated in China. Moreover, no reports have been documented on the effects of various extraction solvents and ultrasound-assisted extraction on the physicochemical properties and bioactivities of polysaccharides from baobab fruit pulp. Therefore, the present study employs three solvents—water, 0.1 M hydrochloric acid, and 0.1 M sodium hydroxide—to extract polysaccharides using hot water stirring extraction and ultrasound-assisted extraction, respectively. The differences among polysaccharides extracted by different methods are then compared via characterization: their chemical composition, molecular weight, monosaccharide composition, FT-IR spectra, conformation, UV absorption, scanning electron microscopy (SEM) images, thermal stability, antioxidant activity, and α-glucosidase inhibitory activity. This provides a theoretical foundation for the potential application of polysaccharides from *Adansonia suarezensis* fruits in pharmaceuticals and functional foods.

## 2. Materials and Methods

### 2.1. Materials and Reagents

*Adansonia suarezensis* fruit pulp was obtained from Jubaolin Planting Professional Cooperative (Zhanjiang, China). Monosaccharide standards including L-Ara, D-Man, D-Gal, D-GluA, D-Glu, D-GalA, L-Fuc, D-Xyl, Rha, along with pNPG (4-nitrophenyl-α-D-glucopyranoside), were sourced from Yuanye Biotechnology Co., Ltd. (Shanghai, China). α-glucosidase was sourced from Sigma-Aldrich Chemical Co., St. Louis, MO, USA. All reagents and chemicals utilized in this study were of analytical or chromatographic grade.

### 2.2. Preparation of ASPs

The shells of *Adansonia suarezensis* fruit were broken, and the pulp was separated from the seeds. Subsequently, the pulp was ground, sieved through a 60-mesh sieve, and dried. With slight modifications to the method of Zhou et al. [14], the pulp was combined with distilled water, 0.1 M sodium hydroxide (pH = 0.31), and 0.1 M hydrochloric acid (pH = 10.83) at a 1:25 (*w*/*v*) ratio, then processed by either hot water extraction with stirring at 65 °C for 1 h, or by continuous ultrasonic-assisted extraction (350 W, 65 °C) for 1 h. After cooling, the mixtures were filtered through 200-mesh gauze to collect the supernatants. The pH of the supernatants from the acid and alkali extractions was adjusted to 7.0. The supernatants were vacuum-filtered and concentrated to 1/3–1/4 of their initial volume. Concentrates were mixed with a five-fold volume of ethanol and stored at 4 °C for 12 h to induce precipitation. The precipitates were collected by filtration through 200-mesh gauze, redissolved in water, and concentrated again by rotary evaporation to remove residual ethanol. The solutions underwent dialysis against water for 72 h using a 3500 Da molecular weight cut-off bag. Finally, the contents were freeze-dried for 72 h to obtain six polysaccharide samples: ASP-HW, ASP-AC, ASP-AL, ASP-HWU, ASP-ACU, and ASP-ALU.

### 2.3. Chemical Composition Analysis

To quantify neutral sugars, the phenol–sulfuric acid method was applied using a D-xylose standard [15]. We employed the meta-hydroxydiphenyl method, with galacturonic acid as the standard, to analyze uronic acid content [16]. Protein content was quantified using the Bradford method, with bovine serum albumin (BSA) serving as the standard [17]. Total phenolic content was quantified via the Folin–Ciocalteu method, using gallic acid as the standard [18].

The monosaccharide composition of ASPs was determined via high-performance liquid chromatography (HPLC) with 1-phenyl-3-methyl-5-pyrazolone (PMP) pre-column derivatization using an Arc HPLC system (Waters, Milford, MA, USA) [19]. A 2 mL sample solution (2 mg/mL) was combined with 2 mL of 4 M trifluoroacetic acid (TFA) in an ampoule bottle, sealed, and hydrolyzed at 110 °C for 8 h. After cooling, the mixture was evaporated to dryness using a rotary evaporator. To fully eliminate TFA, 1 mL of methanol was added and evaporated repeatedly for a total of six cycles and then redissolved the residue in 2 mL of ultrapure water. Subsequently, 0.5 mL of the polysaccharide hydrolysate or standard was combined with NaOH (0.6 M, 0.5 mL) and PMP (0.5 M, 1 mL) methanol solution. The mixtures were derivatized at 70 °C for 100 min. The solution was neutralized with 1 mL of 0.3 M HCl after cooling. The mixture was extracted with 3 mL of chloroform by vortexing. The lower chloroform phase was discarded, and this extraction procedure was repeated five more times (six times in total). The final aqueous phase was passed through a 0.22 µm microporous membrane filter before HPLC analysis. The analysis utilized an Agilent ZORBAX SB-C18 column (5 µm, 4.6 mm × 250 mm) (Agilent, Santa Clara, CA, USA). The mobile phase, consisting of PBS (pH 6.7, 0.02 M) and acetonitrile (83:17, *v*/*v*), was run at a flow rate of 1 mL/min. The temperature of the column was kept at 30 °C. The injection volume was 10 µL, and the detection wavelength was set at 250 nm. The standard curve equation is shown in Table 1.

### 2.4. Molecular Weight Determination (Mw)

HPGPC was employed to assess homogeneity and average molecular weight. Samples at a 5 mg/mL concentration were filtered through a 0.22 μm aqueous microporous filter prior to analysis by U3000 HPGPC (Thermo, Waltham, MA, USA) with a RID-20A detector (Shimadzu, Kyoto, Japan) and a BRT105–103-101 tandem gel column (8.0 mm × 300 mm). The mobile phase was 0.2 M NaCl. The flow rate was set to 0.7 mL/min. The column temperature was 40 °C, and the injection volume was 50 μL [20].

### 2.5. Particle Size Analysis

ASP samples were diluted to 1 mg/mL with deionized water, filtered through a 0.45 μm aqueous phase membrane filter, and subjected to particle size analysis using a NanoBrook Omni analyzer (Brookhaven Instruments, Holtsville, NY, USA) [21].

### 2.6. Ultraviolet Analysis (UV)

An aqueous ASP solution with a concentration of 1.0 mg/mL was prepared for UV-Vis spectroscopy analysis. The absorbance spectrum was measured from 190 to 600 nm using a Cary 60 UV-Vis spectrophotometer (Agilent, Santa Clara, CA, USA) [22].

### 2.7. Fourier Transform-Infrared Spectroscopy (FT-IR) Analysis

FT-IR spectra of the ASPs were recorded on a TENSOR27 spectrometer (Bruker, Ettlingen, Germany) using the KBr pellet method. This involved mixing the sample with KBr at a 1:100 (*w*/*w*) ratio, thoroughly grinding the mixture, compressing it into a pellet, and scanning within the 4000–400 cm^−1^ range [15].

### 2.8. The Congo Red Test

The triple-helical structure of ASPs was investigated using the Congo red method. A solution of ASPs (1 mg/mL) was combined with an equal volume of a Congo red solution at the same concentration. Subsequently, the NaOH concentration was adjusted by adding 5 M NaOH solution to reach final concentrations of 0, 0.1 M, 0.2 M, 0.3 M, 0.4 M, 0.5 M. After 30 min, the UV-Vis absorption spectra were measured between 400 and 600 nm, and the maximum absorption wavelengths (λ_max_) were recorded. A Congo red solution alone served as the negative control, and a mixture of curdlan and Congo red was used as the positive control [23].

### 2.9. Scanning Electron Microscopy (SEM)

The morphology of the ASPs was observed using a scanning electron microscope (JSM-7610F, JEOL, Tokyo, Japan) at 300× magnification. Prior to observation, the dried sample was secured to a stub with a conductive adhesive, lightly blown with a rubber bulb to remove loose powder, and finally coated with a thin layer of gold by sputtering [7].

### 2.10. Thermal Stability Analysis

The thermal stability of ASPs was assessed using a SA449F3 simultaneous thermal analyzer (Netzsch, Selb, Germany). ASPs (10 mg each) were placed in an alumina crucible. The measurements were conducted under a high-purity nitrogen atmosphere at a flow rate of 20 mL/min. The samples were heated from 40 °C to 500 °C at a constant rate of 10 K/min to assess the degradation temperature and weight loss [24].

### 2.11. Antioxidant Capacities Analysis

DPPH• scavenging activity

After mixing equal volume of the ASP solution and the DPPH (0.2 mM) methanol solution, the mixture was allowed to react in the dark for 30 min. Then, the absorbance was measured at 517 nm and recorded as A_1_. The absorbance of a mixture with equal volumes of ultrapure water and DPPH solution was measured and recorded as A_0_ (control). The absorbance of a mixture with equal volumes of the sample solution and absolute ethanol was measured and recorded as A_2_ (sample background). L-Ascorbic acid served as the positive control. The DPPH radical scavenging activity of the samples was determined using Formula (1) [25].

(1) $$\mathrm{DPPH}•\mathrm{scanenging}\;(\%)=1-\frac{\left(A_{1}-A_{2}\right)}{A_{0}}\times 100$$

ABTS•+ scavenging activity

ABTS (7 mmol/L) and PBS (2.45 mmol/L) stock solutions were mixed in equal volumes and stored in the dark for 12 h to generate the ABTS radical cation working solution. This solution was then diluted with PBS (pH 7.4) to achieve an absorbance of 0.70 ± 0.02 at 734 nm. Then, 1 mL of the ASP solution at various concentrations was mixed with 4 mL of the diluted ABTS working solution. After the reaction mixtures were incubated at 25 °C in the dark for 30 min, the absorbance at 734 nm was measured as A_1_. The control absorbance (A_0_) was determined by replacing the sample solution with ultrapure water. The background absorbance (A_2_) was determined by replacing the ABTS working solution with PBS (pH 7.4). L-Ascorbic acid served as the positive control [26]. The ABTS radical scavenging activity was calculated using Formula (2).

(2) $$\mathrm{ABTS}•+\mathrm{scavenging}\left(\%\right)=1-\frac{\left(A_{1}-A_{2}\right)}{A_{0}}\times 100$$

### 2.12. α-Glucosidase Inhibition Activity

The α-glucosidase inhibitory activity was assessed using the method outlined by Tang et al. [27] with slight adjustments. Briefly, 30 μL aliquots of sample solutions (0–1 mg/mL) were combined with 30 μL of α-glucosidase solution (0.2 U/mL). The mixture was pre-incubated at 37 °C for 10 min, then 30 μL of p-NPG solution (10 mmol/L) was added to initiate the reaction. After incubation at 37 °C for 15 min, the reaction was terminated by adding 100 μL of a 0.1 mol/L Na_2_CO_3_ solution. Finally, the absorbance was measured at 405 nm. A control blank was prepared by replacing the α-glucosidase solution with PBS. The α-glucosidase inhibitory activity was determined using Formula (3).

(3) $$\alpha -\mathrm{Glucosidase Inhibition}(\%)=(1-\frac{\left(A_{4}-A_{3}\right)}{\left(A_{2}-A_{1}\right)})\times 1$$

A_1_: the absorbance of the PBS; A_2_: the absorbance of the α-glucosidase + p-NPG, A_3_: the absorbance of the ASP solution; A_4_: the absorbance of the ASP solution + α-glucosidase + p-NPG.

### 2.13. Statistical Analysis

Statistical analysis was conducted on all data using SPSS software (version 27.0). The experiments were carried out in three independent replicates, and the outcomes are reported as mean values ± standard deviation. For statistical evaluation, a one-way ANOVA was first conducted, followed by Duncan’s and LSD post hoc tests to assess specific group differences. A significance level of *p* < 0.05 was established. Figures were generated using Origin software (version 2025).

## 3. Results and Discussion

### 3.1. Yields and Composition of ASPs

Table 2 presents the yields and fundamental chemical compositions of ASPs obtained through various extraction methods. The results showed that different solvent influenced the polysaccharide yield significantly (*p* < 0.05). Among the methods, ultrasound-assisted alkaline extraction achieved the highest yield of 62.5 ± 1.67%, while hot water extraction resulted in the lowest yield of 50.5 ± 0.31%. Ultrasound treatment alone did not exhibit a significant influence on polysaccharides yield. Studies have revealed that acidic extraction can more effectively cleave glycosidic bonds in polysaccharides and increase the production of bioactive low-molecular-weight polysaccharides. Alkaline solutions disrupt hydrogen bonds between hemicellulose and cellulose in plants, enabling the release and conversion of insoluble polysaccharides into soluble forms, thus increasing polysaccharide yield [28]. Consequently, the polysaccharide yields obtained through acidic and alkaline extraction methods are significantly higher than those achieved via water extraction.

The six ASPs exhibited neutral sugar contents ranging from 19.15 ± 0.20% to 20.87 ± 0.69%, and uronic acid contents between 70.91 ± 0.3% and 79.93 ± 1.05%, indicating that all six polysaccharides are acidic. The total phenolic content varied from 2.98 ± 0.08% to 3.93 ± 0.07%, while the protein content ranged from 0.83 ± 0.03% to 4.17 ± 0.07%.

Among them, ASP-HWU and ASP-HW demonstrated higher neutral sugar and uronic acid contents (*p* < 0.05). In contrast, ASP-AL and ASP-ALU showed lower uronic acid contents but higher protein and total phenolic contents (*p* < 0.05). The hydroxide ions in the alkaline solution likely cause the raw material’s cell walls to swell and disrupt component linkages, enhancing the dissolution of proteins and polyphenols [29]. Consequently, this process leads to a reduction in neutral sugar and uronic acid contents, which is consistent with observations reported for polysaccharides extracted from *Coreopsis tinctoria* buds [30] and blackberry fruit [31] using alkaline solutions.

The monosaccharide composition of ASPs was determined by PMP-HPLC method, and the results are presented in Table 3. The findings reveal that all ASPs consist of galacturonic acid, xylose, arabinose, and galactose. Galacturonic acid was the most abundant monosaccharide, with a molar percentage ranging from 67.23% to 72.87%, followed by xylose at 12.26% to 15.82%. Galactose and arabinose were present in smaller quantities, with molar percentages ranging from 7.25% to 8.46% and 6.70% to 8.56%, respectively. The results align with the neutral sugar and uronic acid content, verifying that all ASPs are acidic pectic polysaccharides. Furthermore, different extraction methods only influenced the proportional composition of the individual monosaccharides without altering the types of monosaccharides present in the polysaccharides. This observation differs from the findings reported by Song et al. [8] and Dimopoulou et al. [32], suggesting variations in the monosaccharide composition of baobab fruit from different geographical origins and varieties.

### 3.2. Molecular Weight Analysis

**Figure 1:**
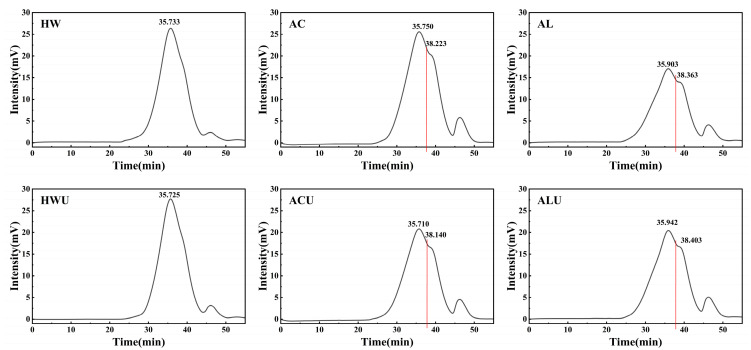
The HPGPC chromatogram of ASPs.

Natural polysaccharides, as a type of biomacromolecule, may have their biological activities and applications influenced to some extent by their molecular weight [33]. Generally speaking, a lower molecular weight typically implies better water solubility, as shorter molecular chains exhibit a stronger capacity to form hydrogen bonds with water molecules and reduced steric hindrance [34]. Consequently, biologically active compounds can be more effectively absorbed and utilized within the organism. The weight-average molecular weights (Mw) of the ASPs, presented in Table 2 and Figure 1, followed a descending order: ASP-HWU (24,750 Da) > ASP-HW (24,686 Da) > ASP-ACU (20,997 Da) > ASP-AC (20,595 Da) > ASP-AL (19,813 Da) > ASP-ALU (19,600 Da). On the HPGPC chromatograms, ASP-HWU and ASP-HW exhibited symmetrical single peaks. In contrast, the acid- and alkaline-extracted polysaccharides showed the presence of additional peaks corresponding to lower molecular weights, which is likely attributable to the cleavage of some polysaccharide chains by the acidic and alkaline solutions, resulting in shorter polymer chains. Based on the monosaccharide composition results, the ASPs are identified primarily as acidic pectic polysaccharides. Alkaline extraction is known to cause more severe disruption to the structure of pectic polysaccharides via β-elimination reactions [35]. In contrast, acid extraction predominantly degrades neutral sugar side chains, while leaving esterified groups on the pectic polysaccharides relatively well preserved [36]. As a result, alkaline-extracted polysaccharides exhibited lower molecular weights and hold better potential for developing liquid functional beverages, oral solutions, or pharmaceuticals requiring rapid release.

The polydispersity index (Mw/Mn) was 1.02 for both ASP-HWU and ASP-HW, while it ranged between 1.15 and 1.16 for the other ASPs. This indicates that although acid and alkaline extraction induced partial degradation of polysaccharide chains, the resulting extracts still displayed a relatively homogeneous molecular weight distribution.

### 3.3. Particle Size Analysis

**Figure 2:**
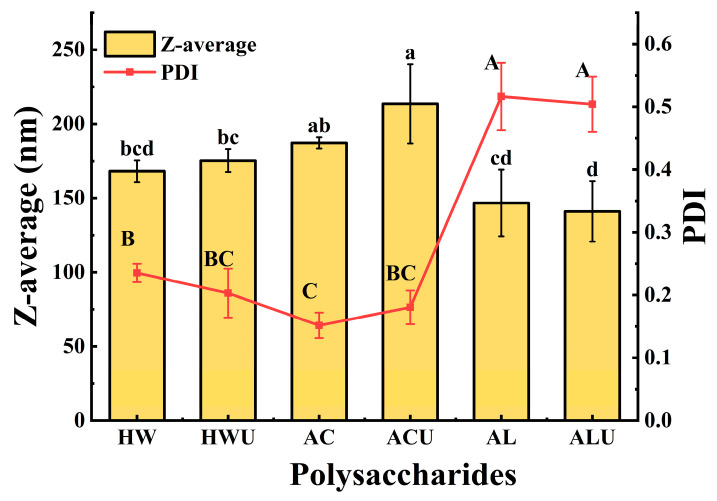
The particle size of ASPs. For the Z-average, values marked with lower letters are significantly different (*p* < 0.05). For the PDI, values marked with upper letters are significantly different (*p* < 0.05).

Polysaccharide particle size often correlates positively with molecular weight, providing an indirect measure of the latter. As shown in Figure 2, the particle sizes of ASPs ranged from 140.97 ± 20.38 nm to 213.53 ± 26.73 nm. ASP-ACU and ASP-AC had particle sizes of 213.53 ± 26.73 nm and 187.20 ± 3.82 nm, respectively, which were significantly larger than the other ASPs (*p* < 0.05). This observation is inconsistent with the molecular weight analysis results. A possible explanation is that the acid-extracted polysaccharides possess stronger intermolecular hydrogen bonding, facilitating increased molecular entanglement and aggregation, thereby forming larger polymeric assemblies [31]. Furthermore, ASP-ACU and ASP-AC also demonstrated the lowest polydispersity index, indicating a more homogeneous particle distribution in aqueous solution.

### 3.4. UV Analysis

**Figure 3:**
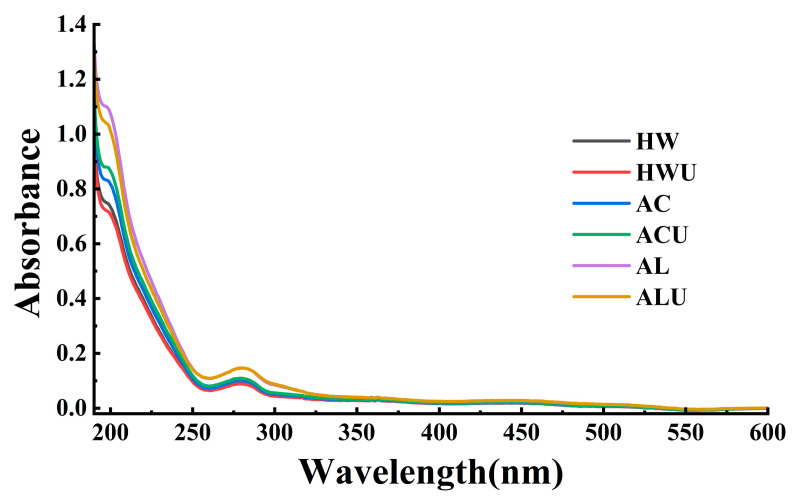
The UV analysis of ASPs.

Characteristic UV absorption peaks can indicate the presence of nucleic acids and proteins in polysaccharides. The UV scanning results of the ASPs are shown in Figure 3. None of the ASPs exhibited an absorption peak at 260 nm, indicating the absence of nucleic acids. In contrast, all samples showed absorption peaks at 280 nm with varying intensities. This observation is the same with the results from the quantitative protein content measurements, confirming the presence of proteins in the ASPs.

### 3.5. FT-IR Analysis

**Figure 4:**
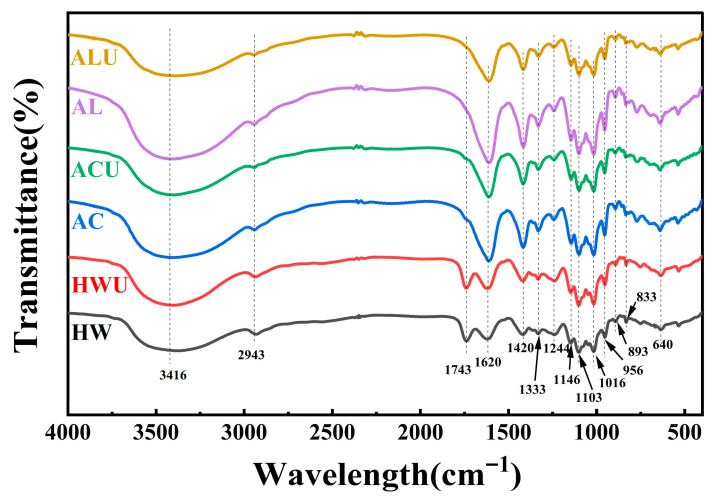
The FT-IR spectra analysis of ASPs.

The characteristic functional groups of ASPs were analyzed through FT-IR spectroscopy. Figure 4 demonstrates that the experimental results revealed characteristic polysaccharide absorption peaks for all ASPs within the 4000–400 cm^−1^ range, with no notable shifts in functional groups. The peak at 3416 cm^−1^ is linked to O-H stretching vibrations, whereas the peaks at 2943 cm^−1^ and 1414 cm^−1^ are associated with C-H stretching and bending vibrations, respectively [37]. The absorption peak at 1743 cm^−1^ in both ASPs-HW and ASPs-HWU is attributed to the symmetric stretching vibration of C=O in ester groups, suggesting esterification. The lack of this peak in alternative extraction techniques is probably due to the cleavage of ester bonds in alkaline conditions, leading to the reduction or loss of C=O vibrations from esterified carboxyl groups (-COOR) [38]. The absorption peak at 1620 cm^−1^ indicates carboxyl group deprotonation (COO^−^), while the -OH out-of-plane deformation vibration at 1333 cm^−1^ verifies uronic acids in the ASPs [39]. The 1244 cm^−1^ peak, attributed to S=O stretching vibrations, signifies the presence of sulfate groups [40]. The absorption peaks in the wavenumber range of 1200–1000 cm^−1^ represent pyranose ring vibrations involving C-O stretching. In addition, the absorption peaks at 1146 cm^−1^, 1103 cm^−1^, and 1016 cm^−1^ are attributed to C-O-H and C-O-C stretching vibrations in the pyranose rings [41]. The absorption bands at 956 cm^−1^ and 640 cm^−1^ indicate β-pyranose configurations in polysaccharides [31]. The peak at 893 cm^−1^ arises from the stretching vibration of β-glycosidic bonds, whereas the 833 cm^−1^ peak is attributed to the stretching vibration of α-glycosidic bonds [14].

### 3.6. Conformational Analysis

**Figure 5:**
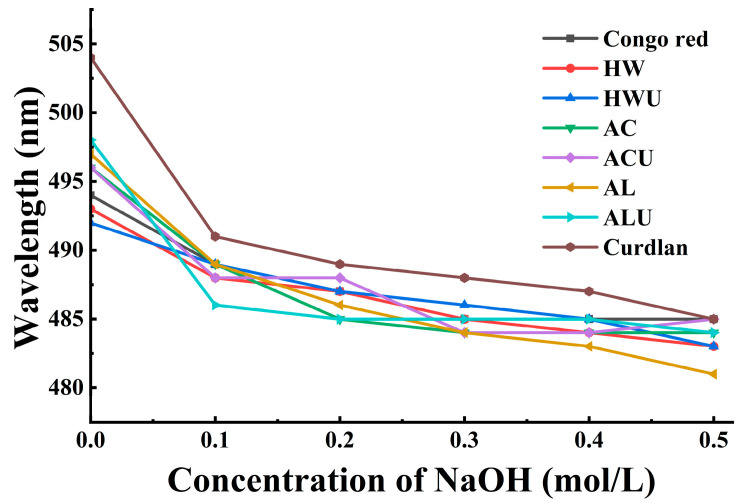
The Congo red experiment results of ASPs.

The bioactivity of polysaccharides is significantly influenced by their triple-helical structure. The observed bathochromic shift in the λ_max_ (maximum absorption wavelength) of the polysaccharide–Congo red complex, relative to Congo red alone, is considered diagnostic of a triple-helix structure [42]. However, as the NaOH concentration increases, the triple-helical conformation of polysaccharides can gradually transition to a single-coil form. This transition diminishes the interaction between polysaccharides and Congo red, resulting in a reduced observed λ_max_. Figure 5 illustrates the λ_max_ values as follows: positive control (curdlan) at 504 nm, ASP-AC at 496 nm, ASP-ACU at 496 nm, ASP-AL at 497 nm, and ASP-ALU at 498 nm. These wavelengths exhibit a notable red shift relative to the Congo red solution (494 nm), suggesting the existence of a triple-helix structure. The λ_max_ values for ASP-HW and ASP-HWU were 493 nm and 492 nm, respectively, indicating no red shift and suggesting the lack of a triple-helical structure in these polysaccharides. The triple helical structure of the ASPs remained stable primarily due to intramolecular and intermolecular hydrogen bonds [43]. Therefore, it is possible that the action of acid and alkaline solutions liberates more active groups, which promotes hydrogen bonding interactions between polysaccharide chains, thereby endowing ASP-AC, ASP-ACU, ASP-AL, and ASP-ALU with a triple-helix structure. Conversely, ASP-HW and ASP-HWU lack a triple-helix structure. This difference may be attributed to the relatively mild conditions of hot water extraction, which likely results in the extraction of pectic polysaccharides with higher molecular weight, more side chains, or a higher degree of esterification. This complex structure may hinder the regular arrangement and tight packing of molecular chains, thereby preventing the formation of a stable triple-helix conformation.

### 3.7. SEM Analysis

**Figure 6:**
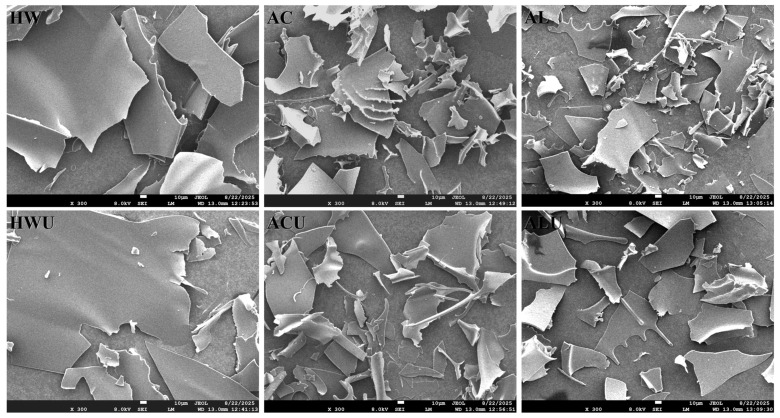
The SEM pictures of ASPs (300×).

Figure 6 presents the SEM pictures of the ASPs. ASP-HW and ASP-HWU exhibited relatively smooth, dense, and irregular large flake-like structures. The flaky structure of ASP-AC and ASP-ACU appeared disrupted and loose, with irregular curls likely resulting from the cross-linking and aggregation of galacturonic acid [31]. In contrast, the structures of ASP-AL and ASP-ALU also presented as flaky overall but were more fragmented and looser, also accompanied by a small number of irregular curls, attributable to the more severe degradation of uronic acid via β-elimination [35].

### 3.8. Thermal Stability Analysis

**Figure 7:**
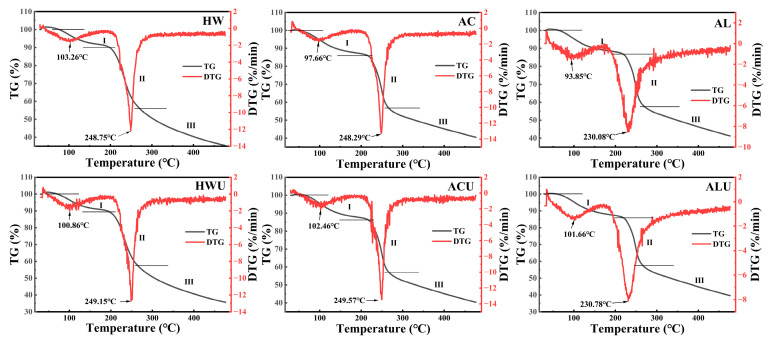
The thermal stability analysis of ASPs. I, II, and III represent different phases of weightlessness.

The thermal stability of ASPs was evaluated by TG and DTG curves measured from 30 to 500 °C, and the results are shown in Figure 7 and Table 4. The TG curves indicated that the thermal decomposition of ASPs occurred mainly in three stages. The initial weight loss mainly results from the evaporation of both free and bound water in the polysaccharides [44]. The weight reduction in the second stage likely results from the cleavage of glycosidic bonds and the degradation of polysaccharide side-chain residues [38]. The third stage of weight loss is attributed to the fragmentation of depolymerized polysaccharide segments. During the initial phase, the weight losses were recorded as ASP-HW (9.89%), ASP-HWU (10.68%), ASP-AC (14.04%), ASP-ACU (13.84%), ASP-AL (13.13%), and ASP-ALU (14.15%). ASP-AL and ASP-ALU exhibited wider temperature ranges for this stage (ASP-AL: 30–219.65 °C; ASP-ALU: 30–219.98 °C), indicating their superior water-binding capacity.

DTG curves, derived as the first derivative of TG curves concerning temperature or time, illustrate the correlation between mass loss rate and temperature or time [21]. The maximum mass loss rate temperature (T_max_) serves as a key indicator of polysaccharide thermal stability, with a higher T_max_ generally reflecting greater thermal resistance [45]. ASP-AL (230.08 °C) and ASP-ALU (230.78 °C) exhibited lower T_max_ values, likely due to the reduced galacturonic acid content in alkaline-extracted polysaccharides [46], resulting in decreased intermolecular cross-linking and thermal stability.

### 3.9. Antioxidant Capacity Analysis

**Figure 8:**
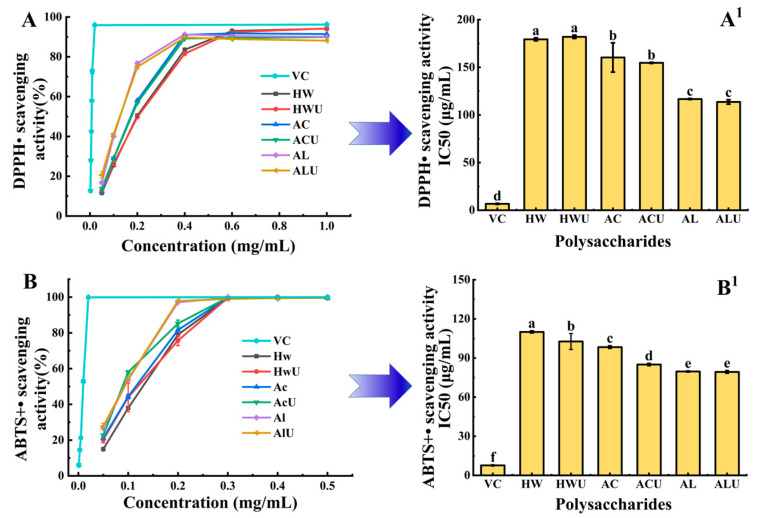
Antioxidant activities of ASPs include DPPH (**A**,**A^1^**) and ABTS (**B**,**B^1^**) radical scavenging activities. For a given parameter, values assigned distinct superscript letters differ significantly (*p* < 0.05).

Figure 8 presents the antioxidant capacity of ASPs extracted via various methods, assessed using DPPH and ABTS radical scavenging assays. DPPH radical scavenging activity depends on the hydrogen-donating capacity of compounds [44]. As shown in Figure 8A,A^1^), ASP-AL and ASP-ALU demonstrated stronger DPPH radical scavenging capacity (*p* < 0.05), with IC_50_ values of 116.67 ± 0.58 μg/mL and 113.67 ± 2.31 μg/mL, respectively. Ultrasound assistance showed no significant effect on DPPH radical scavenging activity. In addition, Figure 8B,B^1^ demonstrates that ASP-AL and ASP-ALU showed significantly higher ABTS radical scavenging activity (*p* < 0.05), with IC_50_ values of 79.67 ± 0.58 μg/mL and 79.33 ± 1.15 μg/mL, respectively, aligning with the DPPH radical scavenging findings. This may be attributed to the lower molecular weight of polysaccharides extracted with alkaline solution, as lower-molecular-weight polysaccharides are often associated with enhanced antioxidant properties, which is ascribed to their stronger intramolecular O–H hydrogen bonding [47]. These findings indicate that alkaline-extracted polysaccharides possess superior antioxidant activity compared to those extracted with water or acid. This observation aligns with previously reported results for polysaccharides from *Coreopsis tinctoria* buds [30] and *Sargassum* [14].

### 3.10. α-Glucosidase Inhibitory Activity

**Figure 9:**
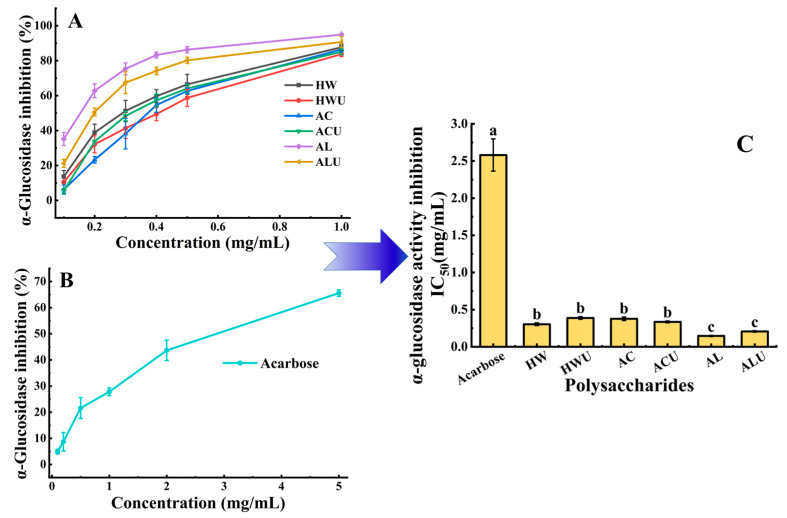
Illustrates the α-glucosidase inhibitory activity of ASPs (**A**) and acarbose (**B**), along with their IC_50_ values (**C**). For a given parameter, values assigned distinct superscript letters differ significantly (*p* < 0.05).

α-glucosidase inhibition activity can delay glucose absorption and represents one of the most effective approaches for managing postprandial hyperglycemia [48]. The results of α-glucosidase inhibitory activities of the ASPs are exhibited in Figure 9. The IC_50_ values of the six polysaccharides, ranked from lowest to highest, are as follows: ASP-AL (0.146 ± 0.01 mg/mL), ASP-ALU (0.206 ± 0.01 mg/mL), ASP-HW (0.303 ± 0.02 mg/mL), ASP-ACU (0.335 ± 0.01 mg/mL), ASP-AC (0.376 ± 0.02 mg/mL), and ASP-HWU (0.388 ± 0.02 mg/mL). All ASPs demonstrated significantly stronger inhibition than the acarbose positive control group (2.58 ± 0.22 mg/mL) (*p* < 0.05), indicating their considerable potential for development as therapeutic agents for type II diabetes. ASP-AL demonstrated superior α-glucosidase inhibitory activity, evidenced by its significantly lower IC_50_ value compared to ASP-AC, ASP-ACU, ASP-HW, and ASP-HWU (*p* < 0.05). Ultrasound-assisted extraction showed no significant effect on α-glucosidase inhibitory activity (*p* > 0.05).

### 3.11. Correlation Analysis

**Figure 10:**
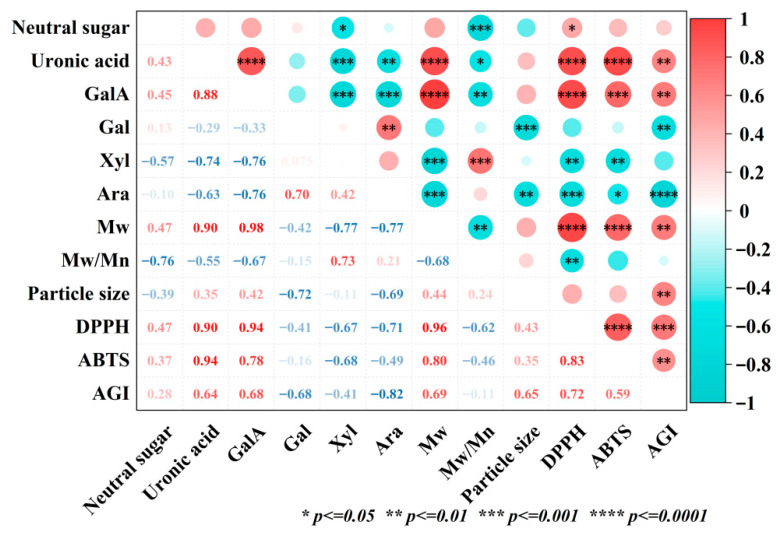
Correlation analysis of ASPs.

To elucidate how the chemical structure of ASPs influences their bioactivity, a correlation heatmap was constructed. (Figure 10). A positive correlation was observed between IC_50_ values for DPPH and ABTS radical scavenging capacities, as well as α-glucosidase inhibitory activity and uronic acid content, indicating that increased uronic acid content corresponded to reduced bioactivity. The carboxyl groups on uronic acids carry negative charges. A high uronic acid content increases electrostatic repulsion between polysaccharide molecules, potentially hindering their approach and interaction with biological targets (e.g., enzymes, free radicals). Alkaline treatment reduced uronic acid content, diminishing this electrostatic barrier and likely altering the charge density and spatial conformation of the polysaccharides, making their active sites more accessible. Moreover, alkaline treatment may enhance overall bioactivity by decreasing esterification, and increasing the RG-I domain proportion in neutral sugars [35]. Additionally, IC_50_ values for DPPH and ABTS radical scavenging, as well as α-glucosidase inhibition, positively correlated with molecular weight, suggesting that lower molecular weights enhance bioactivity. High-molecular-weight polysaccharides often result in increased viscosity, reduced water solubility, and unstable physicochemical properties, potentially restricting their absorption, utilization, and bioactivity. Small polysaccharide molecules possess more free terminal reducing hydroxyl groups and a larger specific surface area, enabling them to donate hydrogen atoms or electrons more efficiently to neutralize free radicals. [49]. Furthermore, lower molecular weight implies smaller spatial dimensions and better membrane permeability, facilitating in vivo binding to targets like α-glucosidase. In summary, the alkaline extraction method synergistically optimizes the molecular characteristics of the polysaccharides by reducing both uronic acid content and molecular weight, thereby maximizing their antioxidant and hypoglycemic activities. This provides clear theoretical guidance for the targeted preparation of highly bioactive polysaccharides from *Adansonia suarezensis* fruit pulp.

## 4. Conclusions

This study systematically assessed the impact of six extraction methods (hot water, acid, alkaline, and their ultrasound-assisted counterparts) on the physicochemical properties and bioactivity of polysaccharides from *Adansonia suarezensis* fruit pulp. The results indicated that alkaline-based extractions (ASP-AL, ASP-ALU) achieved the highest yields (up to 62.47%) and produced polysaccharides with lower molecular weights (19,600–19,813 Da), smaller particle sizes (140.97–146.67 nm), a triple-helix conformation, and superior antioxidant (DPPH IC_50_: 113.67–116.67 μg/mL; ABTS IC_50_: 79.33–79.67 μg/mL) and α-glucosidase inhibitory (IC_50_: 0.146–0.206 mg/mL) activities, outperforming other extracts and the positive control acarbose. Correlation analysis revealed that enhanced bioactivity was associated with lower molecular weight and reduced uronic acid content, which may be attributed to decreased electrostatic repulsion, improved molecular mobility, and enhanced exposure of active sites. Based on these findings, it can be inferred that alkaline extraction not only modifies the structural and compositional properties of polysaccharides but also effectively enhances their functional potential by optimizing molecular conformation and interaction capacity. However, this study is limited to in vitro assays and a specific set of extraction parameters; future research should explore in vivo validation, mechanism of action, optimization of extraction conditions, and comprehensive safety assessments to fully support the potential application of ASPs in nutraceuticals and functional foods.

## Figures and Tables

**Table 1:** Monosaccharide standard curve equation.

Item	Standard Curve	R^2^
Man	y = 7 × 10^6^x + 263,992	0.9897
GlcA	y = 6 × 10^6^x − 684,086	0.9934
GalA	y = 4 × 10^6^x + 1 × 106	0.9991
Rha	y = 6 × 10^6^x − 2 × 106	0.9983
Glc	y = 3 × 10^6^x + 545,556	0.9701
Gal	y = 8 × 10^6^x − 644,473	0.9983
Xyl	y = 7 × 10^6^x + 85,922	0.9892
Ara	y = 9 × 10^6^x − 564,969	0.9757
Fuc	y = 3 × 10^6^x − 44,468	0.9713

**Table 2:** Extraction yield and chemical composition of ASPs. For a given parameter, values assigned distinct superscript letters differ significantly (*p* < 0.05).

Item	HW	HWU	AC	ACU	AL	ALU
Yield (%)	50.53 ± 0.31 ^b^	50.93 ± 3.06 ^b^	59.47 ± 0.42 ^a^	59.60 ± 0.72 ^a^	62.13 ± 0.31 ^a^	62.47 ± 1.67 ^a^
Neutral sugar (%)	20.25 ± 0.61 ^ab^	20.87 ± 0.69 ^a^	19.15 ± 0.20 ^c^	19.17 ± 0.30 ^c^	19.22 ± 0.67 ^c^	19.85 ± 0.16 ^bc^
Uronic acid (%)	79.93 ± 1.05 ^a^	79.59 ± 0.54 ^a^	74.71 ± 0.13 ^b^	74.33 ± 0.21 ^b^	72.69 ± 0.14 ^c^	70.91 ± 0.35 ^d^
Total phenols (%)	3.10 ± 0.11 ^c^	2.98 ± 0.08 ^d^	3.22 ± 0.05 ^b^	3.14 ± 0.04 ^bc^	3.93 ± 0.07 ^a^	3.83 ± 0.05 ^a^
Protein (%)	0.92 ± 0.02 ^d^	0.83 ± 0.03 ^d^	1.52 ± 0.01 ^b^	1.44 ± 0.03 ^c^	4.11 ± 0.03 ^a^	4.17 ± 0.07 ^a^
Monosaccharide composition (molar ratio, %)						
Galacturonic acid	71.30	72.87	69.23	70.29	67.94	67.23
Galactose	8.40	7.25	7.35	7.24	8.00	8.46
Xylose	12.26	13.18	15.68	14.99	15.82	15.75
Arabinose	8.04	6.70	7.74	7.48	8.24	8.56

**Table 3:** Molecular weight distribution of ASPs.

Item	Mw (Da)	Mn (Da)	Mw/Mn
HW	24,686	24,224	1.02
HWU	24,750	24,286	1.02
AC	20,595	17,787	1.16
ACU	20,997	18,239	1.15
AL	19,813	17,203	1.15
ALU	19,600	17,030	1.15

**Table 4:** The TG and DTG Analysis of ASPs.

Item	Phase	Temperature (°C)	Weight Loss (%)
HW	I	30~197.54	9.89
	II	197.54~269.57	34.06
	T_max_	248.75	
HWU	I	30~197.20	10.68
	II	197.20~265.13	31.88
	T_max_	249.15	
AC	I	30~215.67	14.04
	II	215.67~266.41	29.2
	T_max_	248.29	
ACU	I	30~217.23	13.84
	II	217.13~266.85	29.09
	T_max_	249.51	
AL	I	30~219.65	13.13
	II	219.65~270.02	29.38
	T_max_	230.08	
ALU	I	30~219.98	14.15
	II	219.98~266.79	28.42
	T_max_	230.78	

## Data Availability

The original contributions presented in this study are included in the article. Further inquiries can be directed to the corresponding authors.
